# Development of an analytical method for the determination of more than 300 pesticides and metabolites in the particulate and gaseous phase of ambient air

**DOI:** 10.1007/s00216-024-05254-4

**Published:** 2024-04-01

**Authors:** Freya Debler, Juergen Gandrass

**Affiliations:** Helmholtz-Zentrum Hereon, Institute for Coastal Environmental Chemistry, Organic Environmental Chemistry, Max-Planck-Str. 1, 21502 Geesthacht, Germany

**Keywords:** Pesticides, Ambient air, High-volume air sampling, QuEChERS, High-resolution mass spectrometry

## Abstract

Pesticides can enter the atmosphere during spraying or after application, resulting in environmental or human exposure. The study describes the optimisation and validation of analytical methods for the determination of more than 300 pesticides in the particulate and gaseous phases of the air. Pesticides were sampled with high-volume air samplers on glass-fibre filters (GFFs) and glass columns filled with polyurethane foam (PUF) and XAD-2 resin. Comparing different extraction methods, a QuEChERS extraction with acetonitrile was selected for the GFFs. For the PUF/XAD-2 columns, a cold-column extraction with dichloromethane was used. Instrumental determination was performed using liquid chromatography/electrospray ionisation-time-of-flight mass spectrometry (LC/ESI-QTOF) and gas chromatography/electron impact ionisation-tandem mass spectrometry (GC/EI-MS/MS). Recovery experiments showed recovery rates between 70 and 120% for 263 compounds on the GFFs and 75 compounds on the PUF/XAD-2 columns. Semi-quantitative determination was performed for 39 compounds on the GFFs and 110 compounds on the PUF/XAD-2 columns. Finally, 27 compounds on the GFFs and 138 compounds on the PUF/XAD-2 columns could be determined only qualitatively. For the determination of the PUF/XAD-2 samples, signal suppression (LC) or signal enhancement (GC) due to matrix effects were determined. Method quantification limits of the optimised methods ranged from 30 to 240 pg/m^3^ for the target compounds on the GFFs, and from 8 to 60 pg/m^3^ on the PUF/XAD-2 columns. The applicability of the method was demonstrated by means of environmental air samples from an agricultural area in the Netherlands.

**Supplementary Information:**

The online version contains supplementary material available at 10.1007/s00216-024-05254-4.

## Introduction

The global use of pesticides has experienced unprecedented growth over the last decades, driven by the demand for food production and pest management. As a result, in 2021, the global market for pesticides reached 43.3 billion US dollars, underscoring the magnitude of their prevalence in modern agricultural practices [1]. Despite their essential role in crop protection, the widespread application of pesticides has raised multiple environmental and health concerns [2–4].

During and after pesticide application, spray drift, volatilization, and wind erosion facilitate the transfer of these chemicals into the air. Depending on the physicochemical properties of the pesticide, product characteristics, texture of the ground, type of application, and weather conditions, pesticides can end up in the atmosphere. There they can partition between the particulate and gaseous phases and impact distant regions beyond their intended target areas [5–7]. This emphasizes the necessity for an in-depth understanding of the distribution of airborne pesticides and their potential exposure and effects on remote and non-target organisms, including humans and ecosystems.

To assess the presence and concentrations of airborne pesticides, appropriate sampling and analytical techniques are of importance. Adsorbent materials have been widely employed to capture these chemicals during air sampling. Most used adsorbents for the analysis of contaminants in air are polyurethane foams (PUF) and XAD resins for the sampling of the gaseous phase, while glass-fibre filters (GFFs) and quartz-fibre filters (QFFs) are preferred for trapping particle-bound pesticides [8, 9]. Sampling techniques include active and passive air sampling. Most air studies focus on passive sampling, with which a quantitative determination of pesticides cannot be determined. Therefore, this study uses active air sampling with high-volume air samplers [10, 11]. Furthermore, most studies analyse only a few selected pesticides, via the employment of GC-MS/MS methods [8, 9]. Consequently, more polar compounds and some currently used pesticides have often been overlooked in air, with emphasis placed on well-known legacy pesticides, such as organochlorines.

To address these limitations and enhance the capability to detect airborne pesticides, this study compares different extraction methods for the analysis of a broad spectrum of pesticides in air via GC-MS/MS and LC-QTOF analysis. Traditional Soxhlet extraction is an effective method for the extraction of pesticides but is time-consuming and requires substantial amounts of solvent. Typical solvent-based extraction methods of pesticides from air are ultrasonic-assisted extraction (UAE), accelerated solvent extraction (ASE), or microwave-assisted extraction (MAE) [8]. In this study, for the GFFs, also an extraction by diffusion was tested.

Drawing inspiration from the quick, easy, cheap, effective, rugged and safe “QuEChERS” approach utilized in food analysis, the adoption of a more cost-effective and efficient extraction method for the extraction of GFFs is proposed [12]. The QuEChERS method employs reduced solvent volumes, making it more economical and faster, and has demonstrated success in the analysis of PAHs in the particulate phase of air samples [13]. To our best knowledge, this is the first work applying the QuEChERS approach for the analysis of airborne particulate pesticides.

The objective of this study is to optimise and validate the extraction of a wide range of pesticides from GFFs and PUF/XAD-2 columns used in high-volume air samplers. The final extraction methods for both matrices are being applied to eight air samples taken within the scope of the EU project SPRINT at a case study site in the Netherlands.

## Experimental

### Consumables

All standards and reagents used were of the highest purity commercially available. Standards were obtained from Restek (Bellefonte, USA) as standard mixtures (LC Multiresidue Pesticide Kit and GC Multiresidue Pesticide Kit). Compounds not included in these mixtures were purchased from Sigma-Aldrich (Darmstadt, Germany) (2.4-D, clopyralid, dimethenamid-p, fluopicolide, fluroxypyr, imazapic, imazapyr, imazaquin, imazethapyr, MCPA, mecoprop-p, metamitron, napropamide, nicosulfuron, propaquizafop, quinmerac, quizalofop-p-ethyl, thifensulfuron-methyl) and from Dr. Ehrenstorfer (Augsburg, Germany) (allethrin, bentazone, bixafen, chlorimuron-ethyl, cycloxydim, dicamba, dichlorvos, diclosulam, halauxifen-methyl, S-metolachlor, metsulfuron-methyl). Isotope-labelled internal standards of >96% purity were purchased from Dr. Ehrenstorfer (Augsburg, Germany) (metalaxyl-D3, tebuconazole-D6, imidacloprid-D4, carbofuran-D4, metsulfuron-methyl-D3, thiabendazole-D6, trans-cypermethrin-D6, gamma-HCH-D6, chlorpyrifos-D10), Sigma-Aldrich (Darmstadt, Germany) (terbuthylazine-D5, bentazone-D7, dimethomorph-(dimethoxy-D6), MCPA-(methyl-D3)) and Cambridge Isotope Laboratories (Tewksbury, USA) (4.4′-DDE ring 13C12). Injection standard 13C3-caffeine (99%) was purchased from Sigma-Aldrich (Darmstadt, Germany), 13C8-PFOA (99%) was purchased from LGC Standards (Teddington, UK), d-TCEP (>98%) was purchased from Wellington Laboratories, and 13C12-PCB-141 (99%) was purchased from Cambridge Isotope Laboratories (Tewksbury, USA). All solvents were purchased in HPLC grade. Methanol was obtained from Merck (Darmstadt, Germany). Hexane, dichloromethane and acetone were purchased from Promochem (Wesel, Germany), and acetonitrile was purchased from VWR (Darmstadt, Germany). Acetic acid was purchased from Fluka Analytical. For the dispersive SPE, anhydrous magnesium sulphate (>99.5%, Sigma-Aldrich), sodium acetate (>99%, Merck), primary secondary amine (PSA, Agilent Technologies) and Bondesil C18 (Agilent Technologies) were used. Syringe filter with regenerated cellulose and a pore size of 0.2 µm (Whatman, Buckinghamshire, UK) were used for the filtration of sample extracts.

### Selection of pesticides

The analytes considered in the present study were selected from the following criteria: (i) data from farmers on the pesticide application on the case study sites covered within the ongoing H2020 project SPRINT, (ii) known occurrence of pesticides from a study on pesticides in air in Germany [14] and (iii) known occurrence of pesticides in European agricultural soils [15, 16]. The reasons for the criteria above were (i) inclusion of pesticides currently applied on agricultural fields in Europe, (ii) inclusion of pesticides recently detected in the air in Europe and (iii) inclusion of pesticides that could be transported via air (volatilization or wind erosion) or evaporate from soil due to previous pesticide applications. From this information, a preliminary list of pesticides was designed, and standard mixtures of pesticides available on the market including these pesticides were bought. The pesticides included in this list were used for the method optimisation (“Optimised instrumental methods” section). For the method validation and sample analysis, 65 further pesticides were included from the final scope within the EU project SPRINT [17] (“Validation of the final method” and “Summary of optimisation and validation of the final method” sections). Pesticides only included for method validation are tagged in the SI (Table S7 and S8). In total, the initial scope included 468 pesticides, including organochlorines, currently used pesticides, as well as pesticide metabolites, from which 335 substances relevant for the project were included in the method validation.

### Sampling and sample media

Samples for the method validation and breakthrough experiments were taken at the research centre campus in Geesthacht, Germany. The campus is located in a forest area 5 km away from the city centre of Geesthacht and 1 km away from the next agricultural field. Two high-volume air samplers (self-constructed) were deployed in parallel. Samples were taken for seven days with sampled air volumes between 1400 and 2300 m^3^. For the enrichment of pesticides in the gaseous phase of the air, glass columns with a glass frit, a slice of polyurethane foam (PUF, Tisch Environmental, Ohio, USA) and 55 g of Amberlite XAD-2 resin (Supelco, Munich, Germany) were used. The PUF/XAD-2 columns were prepared in a clean lab (class 10.000) and cleaned by Soxhlet extraction with solvents of different polarities for 24 h each. The columns were dried using high-purity nitrogen at a pressure of ~ 1.5 bar and sealed in alumina-coated polypropylene (PP) bags. Glass-fibre filters (GFFs) with a diameter of 15 cm for the analysis of airborne particles were purchased from Macherey-Nagel (Düren, Germany). They were baked out at 450 °C for 6 h, wrapped in aluminium foil and sealed in alumina-coated PP bags.

A subset of air samples collected at an experimental farm in the Dutch case study site, included in the EU project SPRINT campaign [17], was used to determine the applicability of the method. Eight samples were taken between May 2021 and January 2022. Four samples reflected pesticide concentrations during the pesticide application period (May to August 2021), and four samples reflected background concentrations during autumn and winter (October 2021 to January 2022). Exact sampling times and sample volumes are in the SI (Table S6).

### Extraction of pesticides from glass-fibre filters

For the extraction of GFFs, four different extraction methods were compared. These were Soxhlet extraction, ultrasound-assisted extraction (UAE), extraction by diffusion and QuEChERS extraction. For all extraction techniques, three GFFs were spiked with the native and isotope-labelled internal standard mix before extraction.

#### Soxhlet extraction

Soxhlet extraction was performed with 250 mL dichloromethane for 16 h. The resulting extract was divided equally into two aliquots, and each aliquot was evaporated to a volume of 300 µL. To prepare for LC-QTOF analysis, 1 mL of methanol was added to one aliquot, and the extract was evaporated under a gentle stream of nitrogen to 150 µL using a Barkey device (Barkey GmbH & Co. KG, Leopoldshöhe, Germany). During the evaporation step, the walls of the vials were rinsed twice with methanol to minimize potential losses of analytes. The extract was then transferred to an LC vial, and 25 µL of the LC injection standard mix (^13^C_8_-PFOA and ^13^C_3_-caffeine) was added. The volume was adjusted with Milli-Q water to achieve a ratio of Milli-Q/methanol of 30:70%, resulting in a total volume of 583 µL.

For GC analysis, 1 mL of hexane was added to the other aliquot, and the same evaporation steps were performed as for the LC analysis. The resulting extract was transferred to a GC vial, and 20 µL of the GC injection standard (^13^C_12_-PCB-141) was added. Before analysis, both extracts were filtered using a syringe filter.

#### QuEChERS extraction

For the QuEChERS extraction, 7 mL Milli-Q water and 15 mL acetonitrile (ACN) were added to the spiked GFFs. The mixture was shaken head-to-head for 30 min. Afterwards, 5 g of anhydrous magnesium sulphate (MgSO4) and 1.5 g of sodium acetate were added. The tube was then vortexed for 1 min and centrifuged at 3500 rpm for 5 min. For LC-QTOF analysis, an aliquot of 125 µL was transferred to a LC vial, and 25 µL of the LC injection standard mix and 350 µL of Milli-Q water were added. The extract was filtered by a syringe filter (0.2 µm, Whatman) prior to analysis. For the GC-MS/MS analysis, an aliquot of 4.5 mL was transferred into a 15 mL Eppendorf tube, and different steps for the clean-up with a dispersive SPE were tested as described in the “Clean-up of sample extracts” section. For the final d-SPE clean-up, MgSO4, primary secondary amine (PSA) and C18 were added. The tube was vortexed for 1 min and centrifuged at 3500 rpm for 15 min. An aliquot of 3.5 mL was transferred into a Barkey vial and evaporated under nitrogen to 150 µL. A solvent switch was performed by the addition of 150 µL of hexane, vortexing the mixture for 1 min, and transferring the upper hexane phase into a GC vial. 20 µL of the GC injection standard was added before analysis.

#### Ultrasound-assisted extraction

For the ultrasound-assisted extraction, the GFF was transferred into a 50-mL Eppendorf tube, and 30 mL dichloromethane were added. The tube was placed in an ultrasonic bath for 15 min. This step was repeated twice. The three aliquots were combined and divided equally into two aliquots. Each aliquot was evaporated to 300 µL.

For LC-QTOF analysis, 1 mL of methanol was added to one aliquot, and the extract was evaporated to 150 µL. The LC injection standard mix and Milli-Q water were added similar to the Soxhlet extraction, and the extract was filtered with a syringe filter (0.2 µm).

For the GC analysis, 1 mL of hexane was added to the second aliquot and evaporated under nitrogen to 150 µL. 20 µL of the GC injection standard was added, and the extract was filtered with a syringe filter (0.2 µm).

#### Extraction by diffusion

The fourth extraction method, which was tested for the GFFs, was the extraction by diffusion. The GFF was transferred into a round-bottom flask, and 50 mL of dichloromethane were added. The mixture was shaken on a horizontal shaker for 1 min and afterwards soaked for 1 h. This step was repeated twice with soaking times of 30 min. The extracts were combined and then separated equally into two aliquots. Each aliquot was evaporated to 300 µL. The subsequent steps were performed as described for the UAE.

### Extraction of pesticides from PUF/XAD-2 columns

For the extraction of pesticides from PUF/XAD-2 columns, two different methods were compared: Soxhlet extraction and cold-column extraction, both using dichloromethane as extraction solvent. For each extraction technique, three PUF/XAD-2 columns were spiked with the native and isotope-labelled internal standard mix prior to the extraction.

#### Cold-column extraction

For the cold-column extraction, the PUF/XAD-2 columns were filled completely with dichloromethane and soaked for 1 h. This soaking step was repeated twice, with each subsequent soaking duration set at 30 min. Finally, the columns were purged with nitrogen for 1 min at a pressure of 1.5 bar. The resulting extract was divided equally into two aliquots, and the subsequent steps were performed as described in the “Soxhlet extraction” section under the “Extraction of pesticides from glass-fibre filters” section.

#### Soxhlet extraction

Soxhlet extraction of PUF/XAD-2 columns was conducted using 350 mL of dichloromethane for a total extraction time of 16 h. The obtained extracts were divided into two equal aliquots, and each aliquot was evaporated to a volume of 300 µL. The subsequent steps were identical to those described for the Soxhlet extraction of the GFFs (“Soxhlet extraction” section under the “Extraction of pesticides from glass-fibre filters” section).

### Clean-up of sample extracts

For the final QuEChERS extraction method of the GFFs, different options of a d-SPE were tested for the GC analysis. These included a d-SPE using 750 mg MgSO4, 114 mg PSA and 114 mg C18, one with only MgSO4 and PSA, one using MgSO4 and C18 and a clean-up with only using a syringe filter (0.2 µm). For LC analysis, a d-SPE step was not added, which is general practice [12]. Due to high matrix effects, for the final CCE extraction method of the PUF/XAD-2 columns, a clean-up step using a d-SPE with 250 mg MgSO4, 38 mg PSA and 38 mg C18 was compared to a clean-up with a syringe filter for both, GC and LC analysis.

### Instrumental analysis

The instrumental analysis was performed using liquid chromatography (Agilent LC 1290 Infinity II) coupled to a quadrupole time-of-flight mass spectrometer (Agilent QTOF mass spectrometer 6546). As ionisation source, an ESI source (AJS Spray Chamber G1958-65138) was used. The QTOF was operated in All-Ions (AI) mode. For separation, an Acquity HSS T3 C18 column (150 × 2.1 mm, 1.8 µm, Waters) was applied with the following optimised gradient: 5 % B (methanol) for 1 min, rise to 30 % B within 1 min, rise to 100 % B within 25 min, 100 % B for 5 min, 5 % B for 1 min. The flow was set to 0.3 mL/min.

For the GC analysis, two different systems were compared concerning method quantification limits. First, a gas chromatograph (Agilent 7890 B) coupled to a quadrupole time-of-flight mass spectrometer (Agilent 7250) was used. Due to high method quantification limits, this system was compared to a gas chromatograph coupled to a triple quadrupole mass spectrometer (Agilent 7010 GC) for compounds that were included in the pesticide list of the SPRINT project. Both instruments were fitted with a multimode injector (MMI) in pulsed splitless mode. The sample injection volume was 1 µL. The GC was equipped with two HP-5MS columns (15 m × 0.25 mm, 0.25 µm, Agilent Technologies) with mid-point backflush. The MS transfer line and the ion source (electron impact ionisation, EI) were set to 300 °C and 250 °C, respectively. The final oven program was initial 60 °C for 1 min, 10 °C/min to 160 °C and 5 °C/min to 300 °C and held for 5 min.

For the development of the instrumental analysis methods, different parameters were optimised. For the GC-QTOF, the temperature gradient for the GC oven was optimised. For the GC-triple quadrupole instrument, mass transition and the collision energy were determined and optimised. For the LC-QTOF, the gradient of methanol/Milli-Q water was optimised, and the source and mass-spectrometer parameters, namely nebulizer pressure, sheath gas temperature and pressure, drying gas temperature and pressure, fragmentor voltage, nozzle voltage, capillary voltage, and octopole voltage, were optimised.

### Analytical method validation

#### Calibration

For the determination of instrumental detection limits (LODs), instrumental quantification limits (LOQs), and linearity, matrix-matched calibrations (extract of clean PUF/XAD-2 columns or clean GFFs) were used in concentration ranges from 0.05 to 500 pg/µL. Depending on the instrument, 8–10 calibration points were included. The LODs and LOQs were determined by the threefold (LOD) or tenfold (LOQ) signal-to-noise ratio of the calibration samples. Matrix-matched calibration levels of 100 pg/µL were injected ten times to evaluate the instrumental precision.

#### Matrix effects

During the ionisation process of the compounds, matrix effects were observed, resulting in either signal enhancement or signal suppression of the analytes. Interfering matrix constituents originated from sampled air as well as from pre-cleaned GFFs and PUF/XAD-2 columns. Samples with sufficient low pesticide concentrations could not be obtained. Thus, it was decided to use extracts from pre-cleaned GFFs and PUF/XAD-2 columns to compensate at least for these matrix interferences. Therefore, a comparative analysis was conducted between a solvent calibration and a partially matrix-matched calibration for both, LC-MS and GC-MS.

#### Blank experiments

Laboratory blanks were evaluated during the extraction process. Solvent blanks (*n*=3) were determined using 350 mL DCM for the PUF/XAD-2 columns and a mixture of 7.5 mL Milli-Q and 15 mL ACN for the GFFs. The solvents were spiked with the IS mixture and evaporated similarly to the validation samples. Column blanks and GFF blanks (*n*=3) were evaluated by spiking clean columns and GFFs with the IS mixture. Extraction, concentration and determination were done as described above for the respective matrix. During instrumental analysis, solvent blanks (methanol/Milli-Q water 30%/70% on the LC-QTOF and hexane on the GC-QQQ) were measured in between samples.

#### Recovery experiments

Recovery experiments for the target analytes were carried out using spiked GFF and PUF/XAD-2 samples at two different spiking levels, with triplicates for each level and matrix. The GFFs and PUF/XAD-2 columns were sampled for 7 days and spiked with the target analytes and IS mixture prior to the extraction. To identify pesticide concentrations present in the air, for each sampling period of seven days, two GFFs and PUF/XAD-2 columns were sampled. Recovery rates were determined by the internal standard method, allocating an isotope-labelled internal standard to each compound. In total, 14 different isotope-labelled internal standards were used. For compounds, where no direct internal standard was available, the allocation of isotope-labelled internal standards was tested according to the best fit of the following criteria: (i) retention time, (ii) mass and (iii) chemical structure. As method performance acceptability criteria, those described in the guideline SANTE/12682/2019 were used [18]. These included recovery rates between 70 and 120% with a repeatability (RSD) < 20%. As described in the guideline, the method LOQ was calculated as the lowest spike level of the validation meeting these criteria [18].

#### Breakthrough experiments

Breakthrough experiments were conducted for the PUF/XAD-2 columns to check the quantitative collection of the analytes in the gaseous phase. Two sampling columns were operated in series (*n* = 2). The upper column was spiked with the analyte mixture. The same setup was run in parallel without the spike of the analyte mixture. Subsequently, approximately 2000 m^3^ of ambient air on our campus in Geesthacht were drawn through the columns within a sampling period of 7 days. Concentrations of pesticides detected on the second cartridge were determined as breakthrough after the correction with the non-spiked samples taken in parallel. The samples were extracted with the final extraction method for the PUF/XAD-2 columns.

## Results and discussion

### Optimised instrumental methods

Methods for the LC and GC analysis were optimised as described in the “Instrumental analysis” section.

#### Optimisation of chromatographic separation on the LC-QTOF

To optimise the chromatographic separation on the LC-QTOF, several gradient designs (different flow rates and slopes) were tested in both ionisation modes (positive ionisation (PI) and negative ionisation (NI)) in order to obtain a compromise between a good separation of the analytes and matrix constituents and a practical run time. The tested gradients as well as the final parameters are depicted in the SI (Section S1.1–S1.4). The final gradient run time was 35 min with a flow rate of 0.3 mL/min. Compared to other studies that are performing multi-residue analysis for pesticides on the LC, the run time of this gradient was slightly longer. This can be explained with a higher number of compounds that needed to be separated within the run time [16, 19].

#### Optimisation of the ESI ion source settings and MS parameters on the LC-QTOF

The parameters of the electrospray ionisation (ESI) source (nebulizer pressure, sheath gas flow and temperature and drying gas flow and temperature) and the TOF mass spectrometer (capillary voltage, nozzle voltage, fragmentor voltage and octopole voltage), which affect the ionisation of the substances, were optimised for both ionisation modes. Parameters and results for each optimisation step are depicted in the SI (Section S1.2–S1.4).

**Fig. 1:**
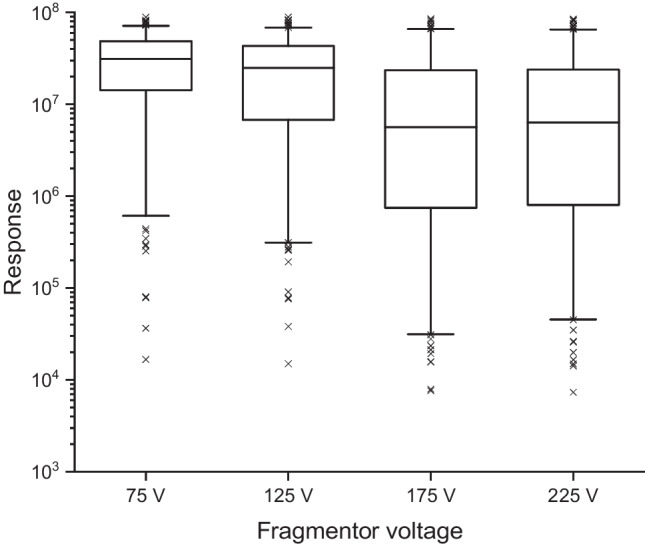
Comparison of different values for the fragmentor voltage in ESI positive ionisation on the LC-QTOF. The box plots reflect the response of 249 analytes. The boxes contain 50% of the data, representing the interquartile range. The upper and the lower end of the box indicate the 75th and 25th percentile. The ends of the vertical lines designate the 5th and 95th percentile. The horizontal bar in the box indicates the median. A cross indicates outliers

The fragmentor voltage had the most significant impact on analyte responses as depicted in Fig. 1. The higher the voltage was set, the lower was the average peak area. Therefore, a low fragmentor voltage of 75 V was chosen for the instrumental method. Table S1.4 (SI) summarizes the final parameters of the instrumental method.

#### Optimisation of chromatographic separation on the GC-QTOF

To optimise the chromatographic separation on the GC-QTOF, different temperature gradients with different start and end temperatures for the oven temperature were tested. The tested temperature gradients as well as the final parameters are presented in the SI (Section S1.5). The final gradient starts at a temperature of 60 °C, runs for 44 min, and ends with a temperature of 300 °C. The final oven temperature settings are described in the “Instrumental analysis” section and the SI (Table S4).

This method was used for the optimisation of the extraction methods. Due to lower sensitivity and high MQLs, the results for the GC-QTOF were compared to a GC-QQQ system. The optimisation and method of the GC-QQQ is described in the following section.

#### Optimisation of MS parameters on the GC-QQQ

For the analysis on the GC-QQQ, the temperature gradient and the temperatures for the ion source and transfer line from the GC-QTOF were transferred to the instrument. Subsequently, mass transitions were determined for 33 compounds included in the pesticide list determined within the project SPRINT that are analysed by GC. Retention times were determined, and collision energies were optimised for these compounds. The final mass transitions and collision energies for each compound can be found in the SI (Table S9). As the GC-QQQ showed better sensitivity and lower MQLs for the analytes, it was used for the analysis of the validation samples and further sample analysis.

### Extraction experiments

For the extraction of the GFFs and PUF/XAD-2 columns, different extraction methods were tested.

#### Comparison of different extraction methods for the extraction of GFFs

**Fig. 2:**
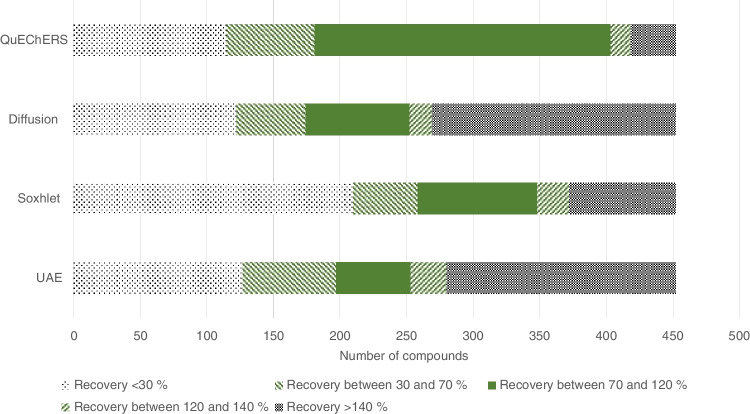
Comparison of relative recovery rates from different extraction methods for the extraction of GFFs. The different bars show the number of compounds detected with recovery rates below 30%, between 30 and 70%, between 70 and 120%, between 120 and 140% and above 140%. UAE = ultrasound-assisted extraction

For the extraction of the GFFs, four different extraction methods were compared. The extraction using the QuEChERS approach together with a d-SPE using MgSO_4_, C18 and PSA as described in the “QuEChERS extraction” section led to the best results in recovery rates and standard deviations. A comparison of all four extraction methods is depicted in Fig. 2. It can be seen that for the QuEChERS extraction, more than 200 pesticides had recovery rates between 70 and 120%, and for more than 300 compounds, recovery rates between 30 and 140% were determined. In comparison, for the other three extraction methods, only about 80 pesticides (diffusion), 90 pesticides (Soxhlet) and 60 pesticides (UAE) had recovery rates between 70 and 120%. Therefore, the QuEChERS method was the preferred extraction method for the GFFs and was used for the method validation and further sample analysis. This is a new approach for the analysis of pesticides in the particulate air phase. Previous studies mostly used Soxhlet extraction with different solvents, depending on the target analytes and instrumental analysis [8, 11]. However, these studies focused on a smaller number of compounds and the QuEChERS approach used in this study showed better recoveries for a high number of analytes compared to Soxhlet extraction.

#### Comparison of different extraction methods for the extraction of PUF/XAD-2 columns

**Fig. 3:**
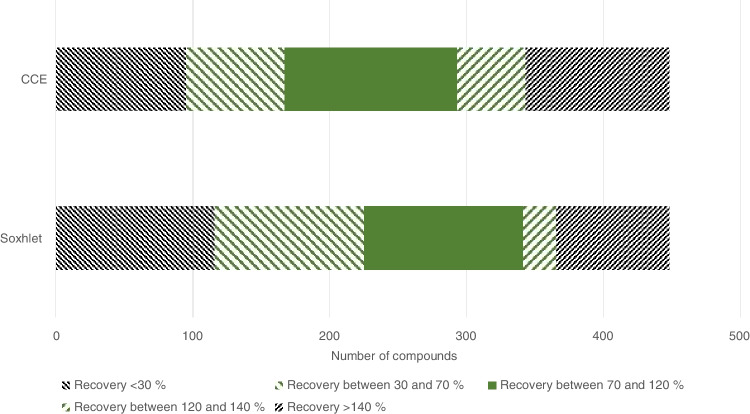
Comparison of different extraction methods for the extraction of PUF/XAD-2 columns. The different bars show the number of compounds detected with recovery rates below 30%, between 30 and 70%, between 70 and 120%, between 120 and 140% and above 140%. CCE = cold-column extraction

For the extraction of PUF/XAD-2 columns, two different extraction methods were compared. Fig. 3 depicts the results of the recovery rates for all compounds. For the cold-column extraction with dichloromethane, more compounds (126) were detected with recovery rates between 70 and 120% compared to the Soxhlet extraction (116). Furthermore, the median recovery rate is closer to 100% (92%) for the cold-column extraction, compared to the Soxhlet extraction (69%). In conclusion, more compounds could be quantified within a range of recovery rates between 70 and 120% with the CCE extraction, and therefore, this method was used for the validation of the method and further sample analysis. Other studies also used dichloromethane as the extraction solvent to determine pesticides in the gaseous air phase [8, 11, 20]. Although these studies focused on Soxhlet extraction or ASE, the cold-column extraction used in this study showed better recoveries and lower matrix effects for the analysis of a high number of target analytes.

### Clean-up of sample extracts

#### Clean-up of GFF extracts for the GC analysis

**Fig. 4:**
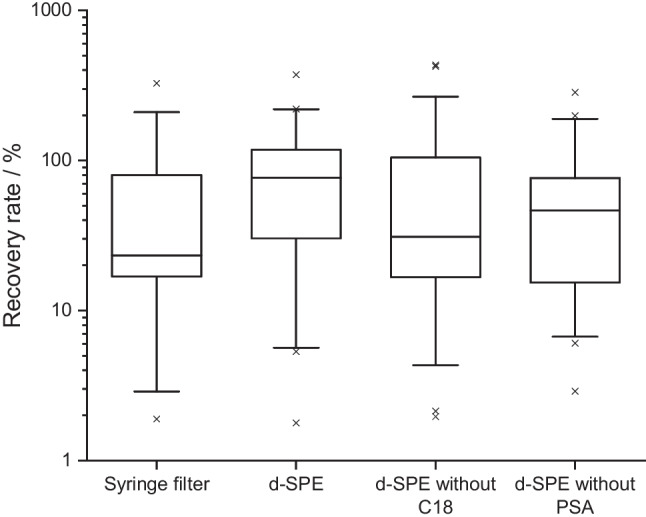
Comparison of different clean-up steps for the extraction of GFFs for the GC analysis. The boxes contain 50% of the data, representing the interquartile range. The upper and the lower end of the box indicate the 75th and 25th percentile. The ends of the vertical lines designate the 5th and 95th percentile. The horizontal bar in the box indicates the median. A cross indicates outliers. d-SPE = dispersive solid-phase extraction; PSA = primary secondary amine

The comparison of different clean-up steps for the GFF extracts for the GC analysis is depicted in Fig. 4. Best recovery rates were determined for the clean-up with a d-SPE using MgSO_4_, PSA and C18 with a median value of the recovery rates at 77%. When the d-SPE was performed without PSA or C18, the median recovery rates were lower and were 31% for the d-SPE without C18 and 46% for a d-SPE without PSA. When only a syringe filter without prior d-SPE was used for the clean-up, the lowest median recovery rates of 23% were detected. Therefore, the clean-up was performed with a d-SPE using MgSO_4_, PSA and C18 for the GC analysis of the validation and real GFF samples. This clean-up step is also in line with other studies using the QuEChERS extraction for other matrices like soil or fish [16, 21].

#### Clean-up of PUF/XAD-2 extracts for GC and LC analyses

**Fig. 5:**
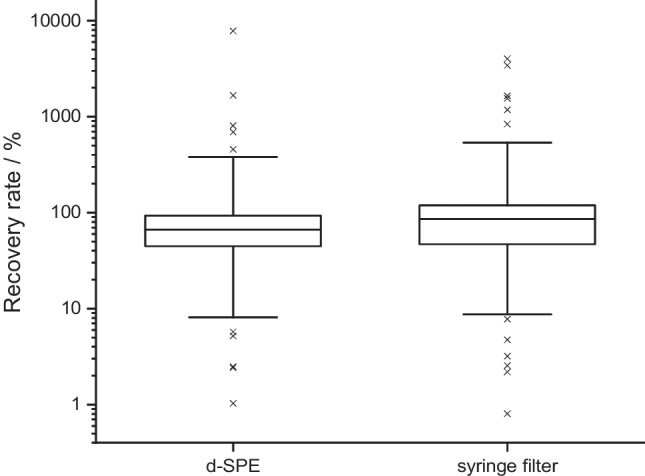
Comparison of different clean-ups for the PUF/XAD-2 columns. The boxes contain 50% of the data, representing the interquartile range. The upper and the lower end of the box indicate the 75th and 25th percentile. The ends of the vertical lines designate the 5th and 95th percentile. The horizontal bar in the box indicates the median. A cross indicates outliers. d-SPE = dispersive solid-phase extraction

The comparison of a clean-up step using a d-SPE with MgSO_4_, PSA and C18 and a clean-up with a syringe filter without prior d-SPE for the extracts of the PUF/XAD-2 columns is depicted in Fig. 5. When using only a syringe filter, the median of the recovery rates is higher (86%) than for the clean-up with a d-SPE (66%). However, values vary less for a clean-up using the d-SPE compared to the syringe filter. When a d-SPE was used, some compounds could not be detected on the LC in PI and NI. For example, the IS MCPA-D_3_ could not be detected anymore in LC/NI. These acidic compounds can be adsorbed to PSA during the d-SPE [22]. Therefore, the syringe filter without prior d-SPE was used for the clean-up during validation and sample analysis. This step is also comparable to other studies using syringe filters for the clean-up of pesticide extracts from the gaseous phase of the air [11]. Especially for the analysis of a broad number of compounds, losses of some compounds during a clean-up step could also be determined by other studies [23].

### Validation of the final method

#### Precision

To evaluate the instrumental precision, the same calibration standard at 100 pg/µL was injected tenfold. The relative standard deviations for most compounds ranged from 0.7 to 18% on the LC-QTOF and from 6 to 16% on the GC-QQQ. For the three target analytes Captan (26%), Folpet (28%), and Triallate (30%), the relative standard deviations were above 20%.

#### Instrumental detection and quantification limits

Instrumental limits of detection (LOD) on the LC-QTOF ranged from 0.05 to 50 pg/µL in positive ionisation and from 0.05 to 50 pg/µL in negative ionisation mode. The instrumental limits of quantification (LOQ) were between 0.05 and 250 pg/µL in LC/PI and 0.1 and 250 pg/µL in LC/NI. For the GC, LODs and LOQs were determined on the GC-QTOF and the GC-QQQ. For the GC-QTOF, LODs were between 0.05 and 250 pg/µL with median values of 5 pg/µL, and LOQs were between 0.05 and 250 pg/µL with median values of 10 pg/µL. For the GC-QQQ, LODs between 0.05 and 5 pg/µL with median values of 0.1 pg/µL and LOQs between 0.05 and 10 pg/µL with median values of 1 pg/µL were determined. Due to tenfold lower LODs and LOQs on the GC-QQQ compared to the GC-QTOF, the validation of the samples was performed with the GC-QQQ instrument.

#### Matrix effects

**Fig. 6:**
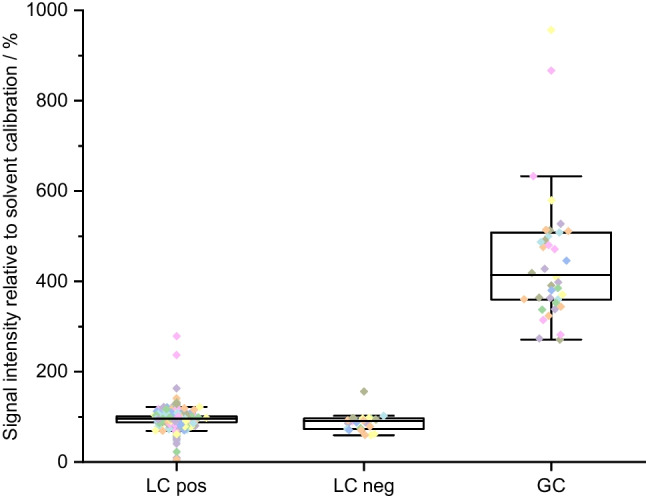
Ratio of signal intensity of a matrix-matched calibration compared to a solvent calibration for the determination of pesticides from GFFs. The boxes contain 50% of the data, representing the interquartile range. The upper and the lower end of the box indicate the 75th and 25th percentile. The ends of the vertical lines designate the 5th and 95th percentile. The horizontal bar in the box indicates the median. The coloured squares represent the recovery rate for each compound

**Fig. 7:**
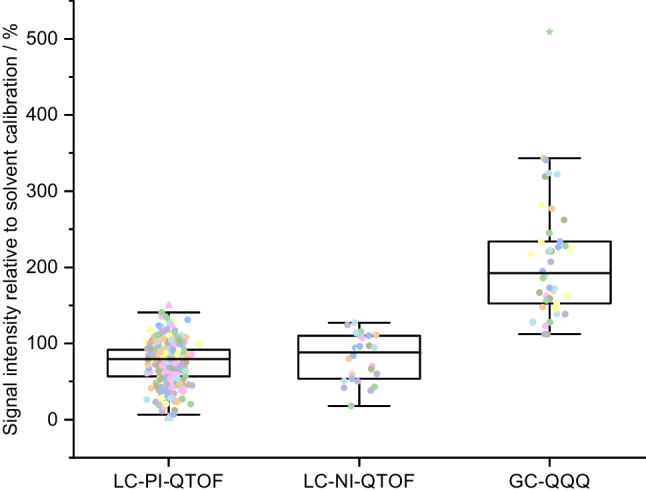
Ratio of signal intensity of a matrix-matched calibration compared to a solvent calibration for the determination of pesticides from PUF/XAD-2 columns. The boxes contain 50% of the data, representing the interquartile range. The upper and the lower end of the box indicate the 75th and 25th percentile. The ends of the vertical lines designate the 5th and 95th percentile. The horizontal bar in the box indicates the median. The coloured squares represent the recovery rate for each compound

The results for the ratios between the partially matrix-matched calibration and the solvent calibration (see the “Matrix effects” section) are depicted in Fig. 6 for the GFFs and in Fig. 7 for the PUF/XAD-2 columns. For GC-MS, the matrix-matched calibration for the PUF/XAD-2 columns exhibited a noteworthy signal enhancement compared to the solvent calibration, with signals showing an increase of signal ratios ranging from 112 to 509% with median values of 192%. For the matrix-matched calibration for the GFFs, signal ratios were between 200 and 1177%, and the median value was 398%. Conversely, in the case of LC-MS, a signal suppression was observed for most analytes for the matrix-matched calibration in both, positive ionisation and negative ionisation mode. For positive ionisation, signal ratios ranged between 0.2 and 149% with median values of 79% for the PUF/XAD-2 columns and between 5 and 279% with median values of 96% for the GFFs. For negative ionisation, the signal ratios for the PUF/XAD-2 columns were between 18 and 127% with median values of 88%. For the GFFs, the ratio between matrix-matched and solvent calibration in the negative ionisation mode ranged from 59 to 156% with median ratios of 91%.

These finding highlight the importance of considering matrix effects and employing matrix-matched calibrations to accurately quantify the analytes of interest in both LC-MS and GC-MS analyses. Such corrections are crucial to obtain reliable and precise results in the presence of matrix interferences [24]. As addressed earlier, the matrix effect occurring from matrix constituents present in sampled air could only be partially included in this study, as a matrix-matched calibration from cleaned GFFs and PUF/XAD-2 columns was used to compensate at least for matrix effects from the sampling material.

#### Recovery experiments

For the recovery experiments, GFFs and PUF/XAD-2 columns were used, which were sampled for 7 days. The recovery experiments were conducted in triplicate at two different concentration levels to assess the accuracy of the analysis. PUF/XAD-2 columns and GFFs were spiked with native and internal standard mixtures. To account for losses during extraction and extract concentration, the analyte areas were normalized to the corresponding internal standard (IS).

For the GFFs, 263 compounds had recovery rates between 70 and 120%. In the case of the PUF/XAD-2 columns, 75 compounds were validated with recovery rates between 70 and 120%. For compounds that did not meet the SANTE criteria (as described in the “Recovery experiments” section), their determination was limited to qualitative assessment. Compounds for which recovery rates between 30 and 140% and relative standard deviations below 40% were detected were classified as “semi-quantitative”. For the GFFs, 39 compounds were determined as semi-quantitative with recovery rates between 32 and 139% and standard deviations between 1.4 and 25%. In case of the PUF/XAD-2 columns, 110 compounds were validated as semi-quantitative, with recovery rates between 32 and 140% and corresponding standard deviations ranging from 1 to 40%.

The applied extraction methods proved to be highly effective for the analysis of pesticides present in the particulate and gaseous phases of the air. Nevertheless, the accurate quantification of compounds, particularly on the PUF/XAD-2 columns, presented notable challenges due to pronounced matrix effects. Despite the adoption of a matrix-matched calibration approach, these effects could not be satisfactorily corrected for certain compounds. Consequently, qualitative analysis emerged as a necessary alternative in these specific cases. When looking at compounds for which an isotope analogue was available as internal standard, most of the compounds for the extraction of GFFs showed recovery rates within the SANTE criteria (10 out of 14 compounds). For metalaxyl, gamma-HCH, imidacloprid and 4.4-DDE, recovery rates for the extraction from GFFs were not satisfactory, even with the use of their isotope analogues. For the PUF/XAD-2 columns, 7 out of 14 compounds met the SANTE criteria. For carbofuran, imidacloprid, gamma-HCH, MCPA, metsulfuron-methyl, tebuconazole and thiabendazole, SANTE criteria were not met. Relative standard deviations between the different validation samples ranged between 30 and 90%, obviously, due to different matrix constituents and effects of PUF/XAD-2 columns cleaned in identical manner. These deviations in precision are also described by other authors for the ESI source, when different batches of the same matrix were used, e.g. for plasma. Niessen et al. referred to this as a “relative matrix effect”, whereas the difference in response between a spiked solvent sample and a spiked matrix-matched sample was referred to as an “absolute matrix effect”. Jemal et al. [25] determined differences in matrix effects for mevalonic acid and its deuterated internal standard in plasma and urine for different batches and also observed differences between the analyte and the appropriate internal standard. To mitigate the impact of matrix effects arising from the PUF/XAD-2 columns, additional clean-up procedures can be explored, or the implementation of a 2D-LC approach may offer a potential solution. Muehlwald et al. [23] compared a d-SPE with the fractionation by 2D-LC for the clean-up of different vegetable matrices and explored better results for the 2D-LC. This knowledge holds promise for improving the accuracy and reliability of quantitative analyses in the presence of challenging matrix effects.

#### Method detection and quantification limits

The method quantification limits (MQLs) are determined by the lowest spiked concentrations for which the compound could be detected. For the GFFs, this resulted in MQLs between 30 and 240 pg/m^3^. For the PUF/XAD-2 columns, MQLs between 8 and 60 pg/m^3^ were determined. MQLs for each compound are depicted in the SI (Table S11 and S12). Compared to other studies, these values are in the same order of magnitude. Coscollà et al. [26] determined 35 pesticides in air samples with MQLs ranging from 2.6 to 75 pg/m^3^.

#### Breakthrough experiments

In the breakthrough experiments with a sampling volume of 2000 m^3^, 12 compounds (carbofuran, pyriproxyfen, chloridazon, cyprodinil metabolite CGA304075, 2.4-DDD, 2.4-DDT, 4.4-DDD, 4.4-DDT, alpha-HCH, delta-HCH, gamma-HCH and chlorpyrifos-methyl) were detected on the second PUF/XAD-2 column. The recovery rates for these compounds on the second column were below 1%, except for gamma-HCH (2%), chlorpyrifos-methyl (4%), 4.4-DDT (35%), 4.4-DDD (11%) and 2.4-DDT (21%), due to their volatile character. For these compounds, possible breakthrough should be considered when analysing real samples.

#### Analysis of environmental samples

Eight environmental samples were taken with a high-volume air sampler close to agricultural fields on a case study site in the Netherlands within the EU project SPRINT. For the samples taken during the pesticide application period between May and August 2021, 35 different pesticides with concentrations ranging from 5 (<MQL) to 670 pg/m^3^ were detected on the GFFs, and 20 different pesticides with concentrations between 6 (<MQL) and 1390 pg/m^3^ were detected on the PUF/XAD-2 columns. In the samples from the sampling period between October 2021 to January 2022, when no pesticides were applied in the adjacent fields, 8 pesticides in concentrations between 12 and 69 pg/m^3^ were detected on the GFFs, and 13 pesticides with concentrations of 2 (<MQL) to 61 pg/m^3^ were detected on the PUF/XAD-2 columns. A figure showing the total concentration of pesticides in each sample is in the SI (Figure S17). The developed method therefore proved applicable for the trace analysis of pesticides in the air in concentrations in the low pg/m^3^ to ng/m^3^ range. Therefore, the method can be applied to determine pesticide background concentrations in air as well as higher pesticide concentrations that occur during pesticide application.

### Summary of optimisation and validation of the final method

An optimised method for the quantification of airborne pesticides in the particulate and gaseous phase of ambient air was developed. For the optimisation of the method, the instrumental parameters on the LC-QTOF and GC-QQQ were optimised. These included the gradient, ion source and MS parameters. A matrix-matched calibration was used for the determination of pesticide concentrations. For the extraction of GFFs, four different extraction methods were compared: Soxhlet extraction, ultrasonic-assisted extraction, QuEChERS extraction and extraction by diffusion. For the PUF/XAD-2 columns, a Soxhlet extraction was compared with a cold-column extraction using dichloromethane. The best results were determined for a QuEChERS extraction of the GFFs and a cold-column extraction with dichloromethane for the PUF/XAD-2 columns. To further improve the recovery rates of the target analytes, different compositions of d-SPE constituents were compared for the GC analysis of the GFFs, and a clean-up with a d-SPE using MgSO_4_, C18 and PSA was tested for the LC and GC analysis of the PUF/XAD-2 columns. For the GFFs, best results were determined when using a d-SPE according to [16] with MgSO_4_, C18 and PSA. However, for the PUF/XAD-2 columns, a clean-up with a d-SPE did not improve the determination of the target analytes for the GC or LC analysis. This shows that the clean-up step according to QuEChERS is not sufficient for the clean-up of DCM extracts of the PUF/XAD-2 columns.

Recovery rates and variability of recovery rates were within the SANTE criteria for 263 compounds on the GFFs and 75 compounds on the PUF/XAD-2 columns. For the remaining compounds, matrix effects leading to signal suppression or signal enhancement influenced recoveries and variability of recoveries. Thus, these compounds could only be determined semi-quantitatively or qualitatively. The applicability of the method was successfully proven by the analysis of ambient air samples from an agricultural site in the Netherlands.

## Conclusions

In this study, a comprehensive method was developed and validated for the determination of more than 300 pesticides in ambient air down to background concentrations in the pg/m^3^ range. High-volume air samplers with GFFs and PUF/XAD-2 columns were applied with sampling volumes of approximately 2000 m^3^. GFFs were extracted with a QuEChERS-based extraction method using acetonitrile, while a cold-column extraction with dichloromethane was used for the PUF/XAD-2 columns.

To the best of our knowledge, this is the first time that the QuEChERS approach was applied for the analysis of airborne particle-bound pesticides. For the instrumental determination, LC-QTOF and GC-QQQ instruments were used. Field samples taken at an agricultural area in the Netherlands demonstrated the applicability of the developed method for background pesticide concentrations down to 2 pg/m^3^ as well as concentrations up to 1390 pg/m^3^ during periods of pesticide application.

Quantitative determination of selected gaseous pesticides analysed with LC-QTOF was impaired due to signal suppression (matrix effects) and caused lower and variable recoveries. Thus, further optimisation of the method should aim at reducing signal suppression by exploration of alternative adsorbent materials and/or extraction solvents. Alternatively, the interference of matrix constituents could be reduced by a clean-up step prior to analysis or by comprehensive (LCxLC) mass spectrometry.

In conclusion, this study serves as a step forward in the analysis of airborne pesticides. The optimised method shows the potential for simultaneous detection and quantification of multiple pesticides in both the particulate and gaseous phase of the air.

### Supplementary Information

Below is the link to the electronic supplementary material.

Supplementary file1 (DOCX 1.01 MB)

Supplementary file2 (XLSX 105 KB)
