# Supplementary material for: Development of an analytical method for the determination of more than 300 pesticides and metabolites in the particulate and gaseous phase of ambient air
Source: Anal Bioanal Chem. 2024 Apr 1;416(12):3059–71. doi: 10.1007/s00216-024-05254-4 (PMC11045619; doi:10.1007/s00216-024-05254-4)
Supplement: Supplementary file 1 — Supplementary file1 (DOCX 1.01 MB) [file 216_2024_5254_MOESM1_ESM.docx]

**Development of an analytical method for the determination of more than 300 pesticides and metabolites in the particulate and gaseous phase of ambient air**

Freya Debler^1^, Juergen Gandrass^1^

^1^Helmholtz-Zentrum Hereon, Institute for Coastal Environmental Chemistry, Organic Environmental Chemistry, Max-Planck-Str. 1, 21502 Geesthacht, [freya.debler@hereon.de](mailto:freya.debler@hereon.de)

**Analytical and Bioanalytical Chemistry**

**Supplemental Information A**

**S1 Methods**

**S1.1 Optimisation of LC gradient**

Table S1:Parameters of tested LC gradients

|  | **Gradient 1** | **Gradient 2** | **Gradient 3** |
| --- | --- | --- | --- |
| **Gradient** | 0 - 1 min : 5 % B | 0 – 1 min: 5 % B | 0 – 1 min: 5 % B |
|  | 1 – 16 min: 100 % B | **1 – 2 min: 30 % B** | 1 – 2 min: 30 % B |
|  | 16 - 21 min: 100 % B | **2 – 27 min: 100 % B** | 2 – 27 min: 100 % B |
|  | 21 – 22 min: 5 % B | 27 – 32 min: 100 % B | 27 – 32 min: 100 % B |
|  |  | 32 – 33 min: 5 % B | 32 – 33 min: 5 % B |
| **Flow** | 0.2 mL/min | 0.2 mL/min | **0.3 mL/min** |

Representative total ion chromatograms of three tested gradients in PI are depicted in Figure S1. The parameters of the tested gradients are listed in Table S1. The figure shows, that the gradient with the shortest run time (A) did not lead to a good separation of the compounds. Therefore, a step of 30% methanol was added and the duration to a solvent ratio of 100% methanol was extended (B). As there were still some compounds, that could not be separated well, the flow rate was changed from 0.2 mL/min to 0.3 mL/min (C). With this gradient, a good separation of the compounds with a short run time could be achieved.


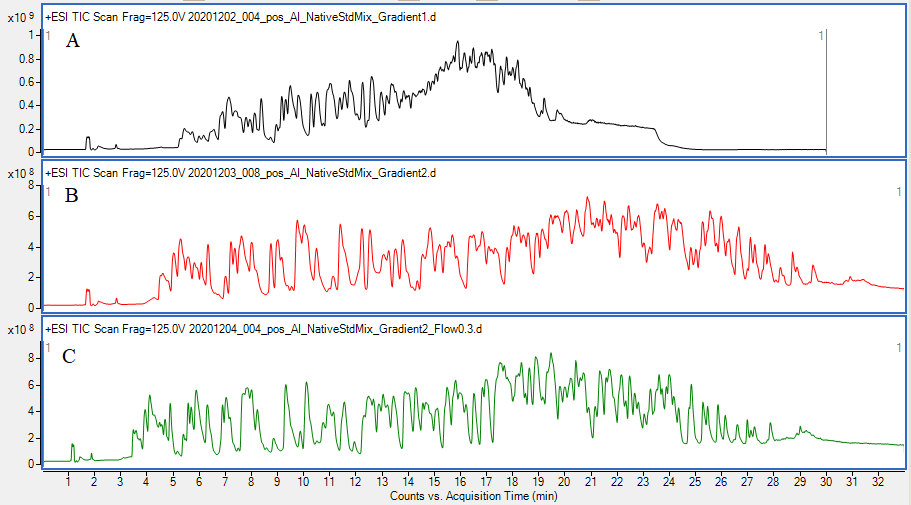


Figure S1: Optimisation of LC gradient in PI

**S1.2 Optimisation of LC-QTOF ion source and MS parameters in positive ionisation**

For all of the following box plots, the boxes contain 50% of the data, representing the interquartile range. The upper and lower end of the box indicate the 75^th^ and 25^th^ percentile. The middle line indicates the median response. The ends of the lines designate the 5 and 95 percentiles. A cross indicates outliers.

Figure S2: Tested parameters of the nebulizer pressure in positive ionisation on the LC-QTOF

Figure S3: Tested parameters of the sheath gas flow and temperature in positive ionisation on the LC-QTOF

Figure S4: Tested parameters of the drying gas flow and temperature in positive ionisation on the LC-QTOF

Figure S5: Tested parameters of the nozzle voltage in positive ionisation on the LC-QTOF

Figure S6: Tested parameters of the capillary voltage in positive ionisation on the LC-QTOF

Figure S7: Tested parameters of the fragmentor voltage in positive ionisation on the LC-QTOF

Figure S8: Tested parameters of the octopole voltage in positive ionisation on the LC-QTOF

**S1.3 Optimisation of LC-QTOF ion source and MS parameters in negative ionisation**

Figure S9: Tested parameters of the nebulizer pressure in negative ionisation on the LC-QTOF

Figure S10: Tested parameters of the drying gas flow and temperature in negative ionisation on the LC-QTOF

Figure S11: Tested parameters of the sheath gas flow and temperature in negative ionisation on the LC-QTOF

Figure S12: Tested parameters of the capillary voltage in negative ionisation on the LC-QTOF

Figure S13: Tested parameters of the nozzle voltage in negative ionisation on the LC-QTOF

Figure S14: Tested parameters of the fragmentor voltage in negative ionisation on the LC-QTOF

Figure S15: Tested parameters of the octopole voltage in negative ionisation on the LC-QTOF

**S1.4 Final parameters of ESI source and MS parameters on the LC-QTOF**

Table S2: Final settings for the source and MS paramters for positive and negative ionisation on the LC-QTOF

|  | Final setting PI | Final setting NI |
| --- | --- | --- |
| Nebulizer pressure / psi | 40 | 35 |
| Sheath gas flow / L/min | 11 | 11 |
| Sheath gas temperature / °C | 350 | 350 |
| Drying gas flow / L/min | 13 | 13 |
| Drying gas temperature / °C | 250 | 250 |
| Capillary voltage / V | 3500 | 3000 |
| Nozzle voltage / V | 0 | 0 |
| Fragmentor voltage / V | 75 | 75 |
| Octopole voltage / V | 400 | 400 |

**S1.5 Optimisation of the GC temperature gradient**

Table S3: Parameters of tested temperature gradients on the GC-QTOF

|  | **Gradient 1** | **Gradient 2** | **Gradient 3** | **Gradient 4** | **Gradient 5** |
| --- | --- | --- | --- | --- | --- |
| **Start temperature** | 60 °C, hold 1 min | 60 °C, hold 1 min | 60 °C, hold 1 min | 60 °C, hold 1 min | 60 °C, hold 1 min |
| **Rate 1** | 5°C/min to 280 °C, hold 5 min | 5°C/min to 300 °C, hold 5 min | 5°C/min to 320 °C, hold 5 min | 10 °C/min to 125 °C, hold 0 min | 10 °C/min to 160 °C, hold 0 min |
| **Rate 2** |  |  |  | 5 °C/min to 300 °C, hold 5 min | 5 °C/min to 300 °C, hold 5 min |

Total ion chromatograms of the tested gradients are depicted in Figure S16. Gradient A, B and C only differentiate between the end temperature. For gradient A, the end temperature was set to 280 °C, for B to 300 °C and for C to 320 °C. This influences especially the compounds that remain on the column for a longer period and only leave it at a high oven temperature. Therefore, an end temperature of 300 °C was chosen, as the compounds were separated well without affecting the column too much with a high end temperature. To reduce the sampling time, a third oven temperature step was included to rise the temperature to 125 °C (D) or 160 °C with steps of 10 °C/min and continue with 5 °C/min to the final temperature of 300 °C. Method E was chosen for further analysis as the compounds were separated best with this method and the run time was still kept as short as possible.


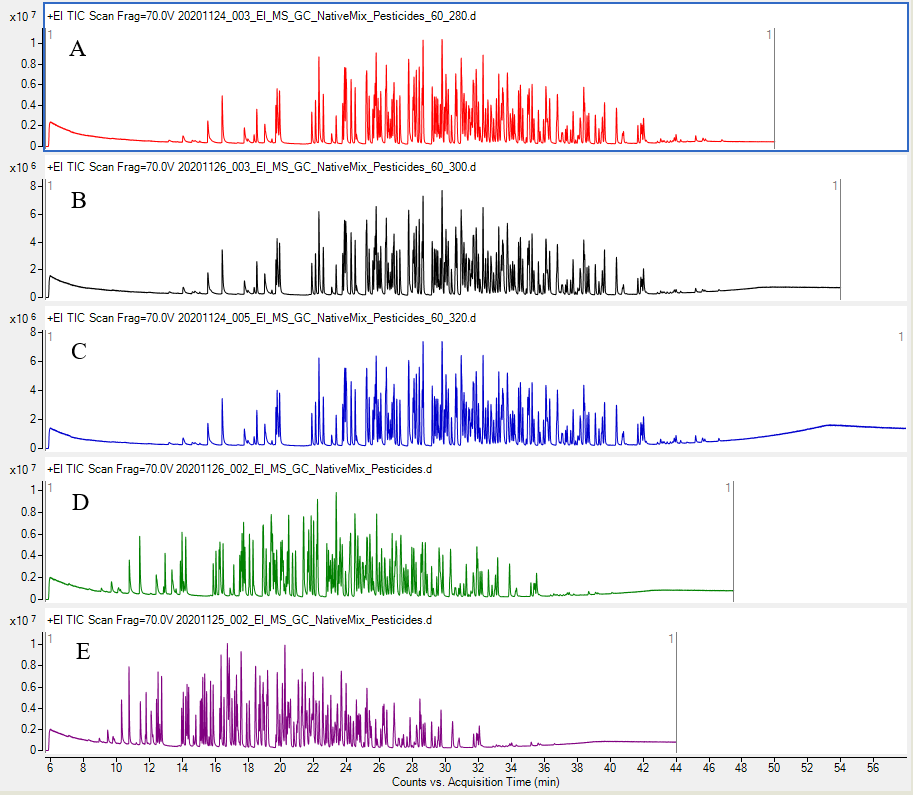


Figure S16: Optimisation of GC temperature gradient

Table S4: Settings of the final GC parameters

|  | Parameter | Setting |
| --- | --- | --- |
| Injector | Injection volume / µL | 1 |
| Oven | Setpoint / °C | 60 |
|  | Hold time / min | 1 |
|  | #1 Rate / °C/min | 10 |
|  | #1 Value / °C | 160 |
|  | #1 Hold Time / min | 0 |
|  | #2 Rate / °C/min | 5 |
|  | #2 Value / °C | 300 |
|  | #2 Hold Time / min | 5 |
| Multimode Inlet | Setpoint / °C | 60 |
|  | Hold Time / min | 0.2 |
|  | #1 Rate / °C/min | 300 |
|  | #1 Value / °C | 300 |
|  | #1 Hold Time / min | 20 |

Table S5: Final parameter settings of the ionisation source and the QTOF on the GC-QTOF

|  | Parameter | Setting |
| --- | --- | --- |
| Ionisation source (EI) | Electron energy / eV | 70 |
| QTOF | Collision energy / V | 0 |
|  | Mass range / amu | 40 - 600 |
|  | Acquisition rate / spectra/s | 8 |
|  | Acquisition time / ms/spectrum | 125 |
|  | Transients/spectrum | 1047 |

**S2. Sampling**

**S2.1 Sampling times and volumes**

Table S6: Air samples taken in the Netherlands

| Sample | Sampling time | Sample volume / m³ |
| --- | --- | --- |
| SPR_NL_2 | 10. – 17.05.2021 | 2322 |
| SPR_NL_6 | 07. – 14.06.2021 | 2425 |
| SPR_NL_11 | 12. – 19.07.2021 | 1494 |
| SPR_NL_15 | 09. – 16.08.2021 | 1324 |
| SPR_NL_24 | 11. – 18.10.2021 | 1774 |
| SPR_NL_28 | 09. – 16.11.2021 | 2196 |
| SPR_NL_32 | 06. – 14.12.2021 | 2298 |
| SPR_NL_36 | 05. – 13.01.2022 | 1411 |

**S2.2 Total pesticide concentration in air samples from the Netherlands**

Figure S17: Total pesticide concentrations in air samples from the Netherlands
